# Editorial: How can corneal biomechanics help with clinical applications?

**DOI:** 10.3389/fbioe.2023.1186938

**Published:** 2023-05-04

**Authors:** Yan Wang, HuaZheng Cao, WeiYi Chen, FangJun Bao, Ahmed Elsheikh

**Affiliations:** ^1^ Tianjin Key Lab of Ophthalmology and Visual Science, Tianjin Eye Hospital, Tianjin Eye Institute, Nankai University Affiliated Eye Hospital, Tianjin, China; ^2^ Nankai University Eye Institute, Nankai University, Tianjin, China; ^3^ School of Medicine, Nankai University, Tianjin, China; ^4^ College of Biomedical Engineering, Taiyuan University of Technology, Taiyuan, China; ^5^ Eye Hospital, Wenzhou Medical University, Wenzhou, China; ^6^ School of Engineering, University of Liverpool, Liverpool, United Kingdom

**Keywords:** corneal biomechanics, corneal refractive surgery, keratoconus, myopia, finite element analysis, clinical application

There is growing interest in corneal biomechanics associated with demands for better precision in clinical diagnosis, treatment of corneal disease and corneal refractive surgery. The cornea possesses a complex balance of stiffness, strength, toughness, and extensibility in order to withstand internal and external forces (Esporcatte et al., 2020). These important properties of the cornea, besides optical characteristics, have often been ignored by clinicians until devices for the *in-vivo* corneal biomechanical assessment were developed. For decades, corneal biomechanical description remained an elusive target until nondestructive testing has taken corneal biomechanics to the clinic and placed it in the hands of clinicians. A deep understanding of corneal biomechanical behavior has critical implications for the management of certain ocular diseases and planning of corneal surgeries. A few examples of the various key clinical applications that highly depend on corneal biomechanical assessment are progression of keratoconus, assessment of corneal refractive surgery, and determination of intraocular pressure (IOP). Recent studies have shown an explosive trend and broadened the scope of clinical translation. Here, we present a Research Topic in this booming field. The topic covers a broad range of themes that reflect the latest progress, most culled from unsolicited original studies and reviews. We hope that this will inspire further enthusiasm for the subject and foster further progress in its clinical applications.

The accurate detection of keratoconus has been widely studied. Tian et al. calculated the corneal elastic modulus according to the relationship between the air-puff forces and corneal apical displacement, and demonstrated that it is more precise than other known dynamic corneal response variables in detecting forme fruste keratoconus. Zhang et al. developed a prognostic nomogram for the diagnosis of subclinical keratoconus. Integrated corneal tomographic and biomechanical assessments achieved significantly better predictability when compared with separate tomographic and biomechanical parameters. Tian et al. reported a gradually decreasing trend in the distance between corneal landmarks (thinnest point, coordinate point of maximum curvature, and corneal apex) with the progression of keratoconus, and the correlation between the three landmark distances and the dynamic corneal response parameters became significant from stage II keratoconus. These results confirmed that the weakening of corneal biomechanical properties from normal to keratoconus might be accompanied by the merging of typical landmark positions.

Based on the current development trend, corneal dynamic deformation in disease diagnosis does not apply to keratoconus only. For example, Ye et al. introduced an air-puff dynamic anterior-segment optical coherence tomography system that was effective in identifying the presence of peripheral anterior synechia in a non-contact approach, avoiding the inconvenience and discomfort caused by traditional indentation gonioscopy. The peripheral cornea is deformed by the air puff jetted from the device, and pressure is transferred to the anterior chamber angle through the aqueous humor. This approach shows great potential for guidance in the diagnosis of angle-closure glaucoma. It is noteworthy that most dynamic corneal response parameters are more significantly correlated with IOP than corneal thickness, as reported by Ye et al., who determined the effect of IOP fluctuation caused by mydriasis on corneal biomechanical metrics. Therefore, changes in corneal thickness and IOP should be considered when evaluating corneal biomechanical changes during disease progression.

Corneal biomechanics is coming of age as an important consideration in surgical procedures designed to change corneal shape and in cross-linking treatments aimed at stiffening the cornea and halting disease progression in keratoconus and iatrogenic ectasia. Xin et al. compared the corneal biomechanical response to transepithelial photorefractive keratectomy (tPRK), femtosecond laser-assisted *in situ* keratomileusis (FS-LASIK), and small-incision lenticule extraction (SMILE), in a population matched for age, sex, corrected refraction, corneal thickness, optical zone, and IOP. The SMILE procedure led to less corneal biomechanical degradation than FS-LASIK, but more than tPRK, in cases with comparable corneal thickness loss. This is in agreement with previous studies reported by our team, indicating that the FS-LASIK procedure induced a greater reduction in corneal tensile strength than SMILE, providing robust evidence for the selection of refractive surgery (Wu et al., 2014; Ma et al., 2018). In a prospective study, Sun et al. reported that topography-guided transepithelial-accelerated corneal collagen crosslinking is effective and safe in correcting low refractive error in keratoconus treatment, as more than 30% of the patients presented an enhanced UCVA by more than three lines, and all topographies showed no sign of disease progression at each examination. On the basis of long-term observations, Tian et al. reported improved CDVA, increased corneal thickness, stable keratometry, and posterior corneal elevation after accelerated transepithelial corneal crosslinking for post-LASIK ectasia during a follow-up period of 2 years.

The current Research Topic covers the corneal biomechanical changes in myopia, which have been viewed as an interesting biomechanical problem. Liu et al. found a lower stress-strain index (SSI), a biomechanical parameter representing corneal material stiffness, in severely elongated eyes (axial length ≥ 26 mm) than in moderately elongated eyes (axial length < 26 mm), and the SSI was negatively correlated with axial length in the former group, but not in the latter group. These results have provided insights into the different eye growth patterns in lower myopia and higher myopia, as axial length carries information about the degree of ocular myopia. Nevertheless, owing to the different refractive powers of the refractive system between individuals, myopic refraction may vary for the same axial length. The ratio of axial length to corneal radius of curvature (AL/CR) is likely to be reliable for the quantitative description of myopic refraction (Scheiman et al., 2016). A retrospective study by Chu et al. showed a negative correlation between SSI and AL/CR regardless of the degree of myopia; however, SSI did not decrease with the worsening of myopia; rather, it exhibited gradual stability at a low level. These findings validate the possibility of assessing dynamic changes in ocular wall stiffness based on corneal biomechanical measurements during the development of myopia.

This Research Topic collects several kinds of plausible, capable technologies in a challenging field and *in vivo* characterization of the corneal constitutive mechanical composition, as the current devices in clinical practice do not yet provide classic biomechanical properties, such as the tangent modulus, with the exception of the SSI. Taking the natural frequency of corneal tissue as a biomarker for corneal biomechanics, Lan et al. confirmed the possibility of micro-forced optical coherence elastography to characterize the global and local features of heterogeneous samples, which can resolve local variations in tissue structure, complementary to current clinical biomechanical assessments. Zhang et al. calculated the corneal strain energy using the viscoelastic strain energy density function and the first-order Prony relaxation function with the first-order Ogden strain energy of the cornea. After completely considering the work done by the air puff as the strain energy of the cornea in attaining whole-corneal displacement, the evaluation of corneal viscoelastic properties *in vivo* was performed by applying an optimized genetic algorithm. Yousefi et al. reported that the experienced load of the cornea during the concave phase of the Corvis test was significantly altered by dynamic changes in the corneal shape, implying a subject-specific loading fluctuation even if the air puff itself was identical. Stiffer corneas showed the least sensitivity to a change in load, whereas more compliant corneas exhibited higher sensitivity. This is important when comparing the same eye after a surgical procedure or topical medication that alters corneal properties. Song et al. linked the elastic modulus of the human corneal stroma measured by tensile testing with optical coherence tomography imaging (*ex vivo*) to corneal deformation measurements using visualization Scheimpflug technology (*in vivo*). They found that a low strain tangent modulus was accompanied by a larger A1 length and A1 deflection area. This indicated the relationship between corneal dynamic response parameters and their intrinsic elasticity, which is helpful in understanding the nature of the measurements produced and the compromises that had to be made to allow nondestructive biomechanical assessment to optimize clinical interpretation.

One of the most useful tools for the simulation of corneal biomechanical response is finite element analysis. In the last two decades, corneal mechanical behavior under different conditions has been discussed using numerical simulation methods, providing theoretical support for clinical practice (Fang et al., 2020a; Fang et al., 2020b). In this Research Topic, Qin et al. propose a method to determine corneal biomechanical parameters based on Ocular Response Analyzer measurements. Redaelli et al. developed a high-fidelity fluid-structure interaction simulation to virtually apply a defined air pulse to a 3D idealized eye model comprising the cornea, limbus, sclera, lens, and humors, and explained the correct variation of IOP during a non-contact tonometry test. Moreover, in order to evaluate biomechanical differences between LASIK and SMILE procedures, Wang et al. applied the iterative algorithm to retrieve the stress-free state of the intact corneal model, LASIK model, and SMILE model. To improve corneal biomechanical stability, it is recommended to create a corneal cap rather than a corneal flap. Using finite-element modeling during orthokeratology, Wu et al. reported that the target myopia reduction had the greatest effect on the central corneal stress value, followed by corneal curvature, while the opposite results were observed in the peripheral corneal area. The authors suggested considering the orthokeratology lens design and the lens fitting process in clinical practice, especially for patients with high myopia and steep corneas.

Another exciting aspect is the cellular and molecular exploration of corneal biomechanics. As reviewed by Yang et al., different types of corneal cells can perceive and transduce mechanical signals in distinct ways, which modulate the expression of specific genes and influence diverse biological functions. These mechanical cues include substrate stiffness, shear stress, and tensile and compressive forces. The mechanisms by which corneal cells interact with different mechanical microenvironments determine many major biological functions under physiological and pathological conditions, which enable physicians to develop therapeutic strategies for corneal disorders from a mechanobiological perspective.

In summary, corneal biomechanics has blossomed from a predominantly academic discipline to a translational enterprise throughout its two-decade history, and we are on the brink of a new era of biomechanics-based intervention. The study on the early diagnosis of keratoconus has attracted the greatest readership, and this is a good example that motivates our efforts to explore, build, and develop this promising field. Further studies on corneal biomechanics will continue to develop methods and measuring parameters. More profound investigation and daring attempts are necessary to build a bridge to clinical application, as well as more accurate and effective diagnosis. The ultimate goal is to optimize the surgical design and guide for disease treatment, and such a future now appears within reach.
